# Developing Value-Orientations in Family Therapy Trainees: A Three-Year Investigation

**DOI:** 10.5964/ejop.v14i3.1519

**Published:** 2018-08-31

**Authors:** Claude-Hélène Mayer, Rudolf M. Oosthuizen

**Affiliations:** a Department of Management, Rhodes University, Grahamstown, South Africa; b Institut für Therapeutische Kommunikation, Europa-Universität Viadrina, Frankfurt (Oder), Germany; c Department of Industrial and Organisational Psychology, UNISA, Pretoria, South Africa; Department of Psychology, Webster University Geneva, Geneva, Switzerland; The Ohio State University Wexner Medical Center, Columbus, OH, USA

**Keywords:** value-orientation, Schwartz value theory, family therapist trainees, Germany, qualitative investigation

## Abstract

Family therapy has advanced as an important therapeutic approach in Europe and, in Germany, future family therapists enter a three-year-training programme every year. Family therapist trainees (FTTs) have hardly been studied in terms of their value-orientations (VOs) and how they see the world. This study aims at researching the value-orientation developments in FTTs during the three-year training period and based on the Schwartz value model. A longitudinal study was carried out over a three-year period. The sample consisted of 65 FTTs. VOs were investigated using open questions in self-developed questionnaires. The findings show that FTTs focus mainly on VOs in the Schwartz value domain of "benevolence", followed by "self-direction" and "universalism". This shows that the well-being of others is in the centre of interest in FTTs. However, VOs which indicate a freedom to make decisions and be self-directed are also extremely important. The least-mentioned value domains include "power" and "stimulation", showing that FTTs are neither focused on status, wealth or richness, nor on excitement or a varied life. With regard to the Schwartz value dimensions, the dimension of "self-transcendence" was the most frequently mentioned, followed by "conservation", "openness to change" and "self-enhancement". Self-transcendence includes the value domains of benevolence and universalism and shows that the preservation and enhancement of welfare of other individuals are highly important, as well as VOs such as understanding, appreciation, tolerance and protection of human beings and the environment. So-called collectivist VOs seem to be more important to German FTTs than individualistic VOs: they focus on the needs of the social group and their VOs show that the systemic view is inherent in their social VOs. Findings further show that FTTs develop their professional identity while consciously strengthening their VOs. This study contradicts previous research which claims that FTs are, to a large extent, unconscious in respect of their VOs. The study shows that FTTs are aware of their VOs and this supports them in facilitating client-centred approaches and develop themselves as FT professionals. Recommendations for future research and practice are provided.

Research on value orientations (VOs) follows a long tradition within the psychological disciplines (Bond, 1998; Jambrak, Deane, & Williams, 2014; Rokeach, 1973; Schwartz, 1994; Schwartz & Bardi, 2001). Discourses on VOs are particularly important in educational and training contexts (Boness, 2002; Patel, 2016) as they determine the individual's interest in self and others (Howell & Larsen, 2015). VOs, as key players in educational and training contexts, have a major underlying impact on the individual's development (Mayer, 2008), particularly in their future roles as therapists and in their professional fields in psychosocial settings (Rusk & Waters, 2015).

VOs are essentially dialectic and contradictory (Hiratsuka, Suzuki, & Pusina, 2016) and the experiences of differences in value-sets often lead to conflictual situations (Barsky, 2014; Berkel, 2005; Janssen & Van Berkel, 2015). The conscious awareness of VOs, however, increases openness and willingness to talk and to cooperate (Druckmann, Broom, & Korper, 1988) and provides the therapist with a more conscious approach and more clarity in therapy (Edwards, 2014), whilst at the same time impacting positively on the development of a professional identity (Levy, Shlomo, & Itzhaky, 2014). VOs in therapy also impact on the therapist-client relationship and the treatment outcomes (Huang & Zane, 2016), whilst building the primary foundation of motivation in individuals and contributing to general mental health and well-being (Jambrak, Deane, & Williams, 2014).

## Value-Orientations and Value Domains and Dimensions

Since the 1970s, research on VOs has gained momentum and, based on Rokeach's (1973) value research, Schwartz and Bilsky (1987) and Schwartz (1994) developed a value-model, which posed the question of a "universal theory of human values". VOs have been defined differently and broadly in recent decades (Alazaizeh, Hallo, Backman, Norman, & Vogel, 2016; Mayton, Ball-Rokeach, & Loges, 1994; Schwartz & Bilsky, 1987, p. 551). However, Schwartz, with his colleagues, has developed an internationally very well recognised and established value model and defined values clearly as follows: "Values are concepts or beliefs about desirable end states or behaviours (terminal and instrumental values), which transcend specific situations; guide the selection or evaluation of behaviour; or order events according to relative importance". Important in this definition is that values are established over time and that they express desirable states or behaviours. They might occur in specific situations; however, they transcend and go beyond these situations. Based on values, priorities are set and behaviour is influenced. For our research, this definition is highly usable, because it defines values specifically, but is at the same time open and broad enough to provide enough freedom for the participants to define values within the Schwartz value model.

Kluckhohn and Strodtbeck (1961) explain that values are cognitive representations and that they are comprised of three forms of universal human sources, namely needs of individuals as biological organisms; requisites of coordinated, social and interpersonal interaction; and security of functions concerning the well-being and survival of groups. VOs are formed through socialisation and thereby become socio-cultural concepts of individuals, groups and societies (Schwartz, 1994); they are universal in their structure, but differ in individuals across countries (Schwartz, 2012).

Values are desirable trans-situational goals, varying in importance, that serve as guiding principles in the life of a person or other social entities (Schwartz, 1994). Implicit in this definition of values as goals is that (1) they serve the interests of some social entity (e.g. groups, family, communities); (2) they can motivate action (behaviour) – giving it direction and emotional intensity; (3) they function as standards for judging and justifying action; and (4) they are acquired both through socialisation to dominant group values and through the unique learning experience of individuals. Schwartz and Bilsky (1987, pp. 551) and Schwartz (1994, pp. 19) define ten universal value domains, including the motivational domains of power, benevolence, universalism, achievement, hedonism, self-direction, tradition and universalism (Sinding, Waldstrom, Kreitner, & Kinicki (2014) (see Table 1). Within the value domains, several values are included. Table 1 provides examples of values in value domains according to Schwartz (1994).

**Table 1:** Motivational Domains – Schwartz Value Model (Schwartz, 1994, p. 22)

Definition and aim of value domains	Exemplary values
**Power:** Social status and prestige, control or dominance over people and resources	Social power, authority and wealth
**Achievement:** Personal success through demonstrating competence according to social standards	Successful, capable, ambitious
**Hedonism:** Pleasure and sensuous gratification for oneself	Pleasure, enjoying life
**Stimulation:** Excitement, novelty, and challenge in life	Daring, varied life, exciting life
**Self-direction:** Independent thought and action-choosing, creating, exploring	Creativity, sistent, compare creativity and successful, curious, freedom
**Universalism:** Understanding, appreciation, tolerance and protection for the welfare of all people and for nature	Broad minded, social justice, equality
**Benevolence:** Preservation and enhancement of the welfare of people with whom one is in frequent personal contact	Helpful, honest, forgiving
**Tradition:** Respect, commitment and acceptance of the customs and ideas that traditional culture or religion provide	Humble, devout, accepting my “portion in life”
**Conformity:** Restraint of actions, inclinations, and impulses likely to upset or harm others and violate social expectations or norms	Politeness, obedience Honouring parents and elders
**Security:** Safety, harmony and stability of society, of relationships and of self	National security Social order, cleanliness

**Figure 1:**
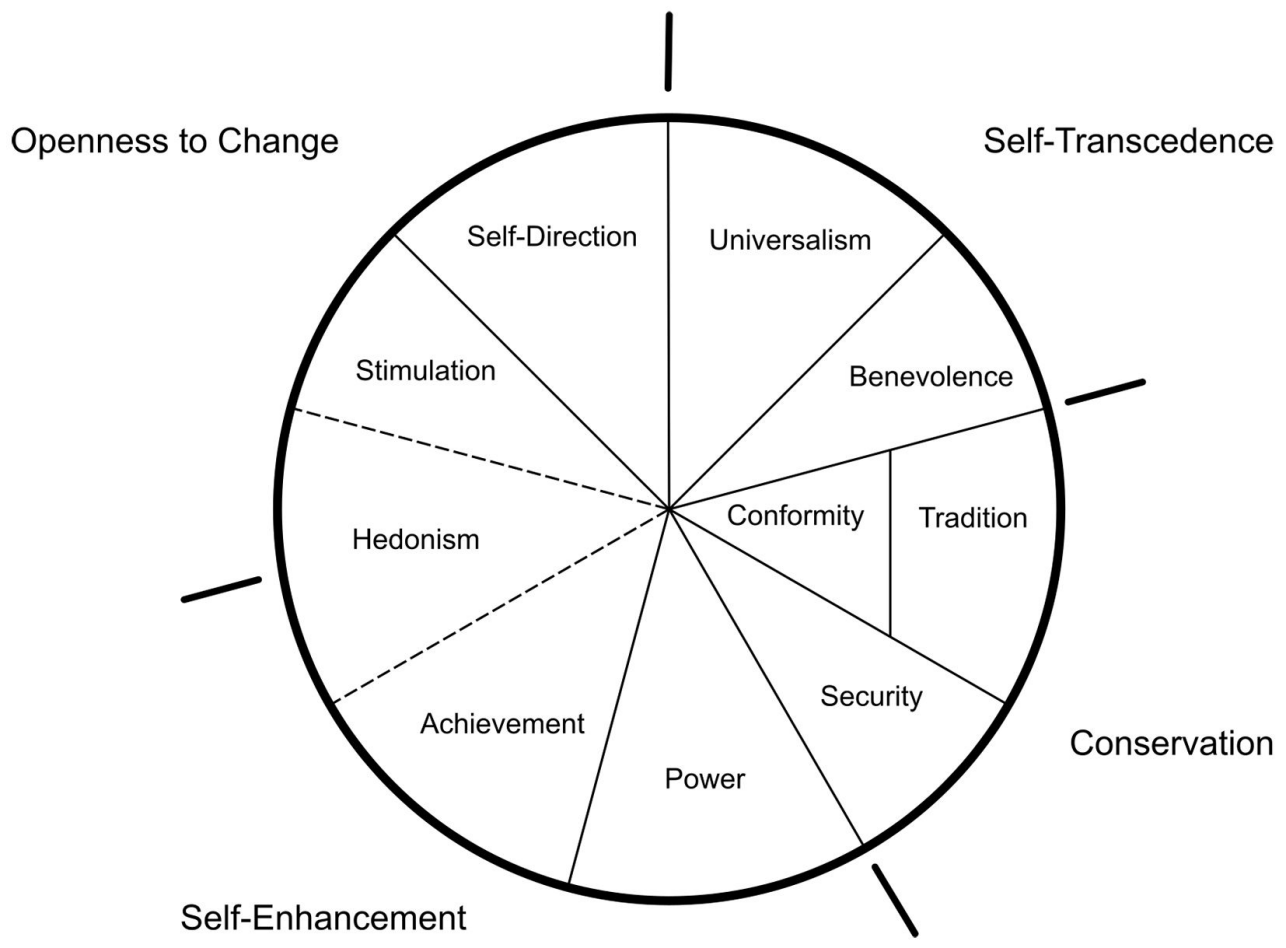
Dimensions and domains – Mayer (2001) adapted from Schwartz Value Model (Schwartz, 1994, p. 24-35)

These value domains have been supported by various research projects (Fromm, 2016; Mayer, 2001; Schwartz & Bardi, 2001) and it has been pointed out that values are conceptions of higher order goals, serving individualistic (achievement, enjoyment, self-direction) or collectivistic (traditional, conformity) goals, which might be interwoven (Oishi et al., 1998) and which are dynamic and changeable (Gouveia, Vione, Milfont, & Fischer, 2015). Values are connected through dynamic interactions and are dependent on each other. Value domains can be classified according to four value dimensions. Schwartz defined four dimensions, including ten value domains. The domains are integrated parts of the dimensions and, as shown in Table 2, Schwartz (1994) has referred specific value examples to specific value domains. Bordering value domains are more transparent than value domains that are in opposition, as described by Schwartz (1994, p. 24-25, see Figure 1): (1) *openness to change*: independent thoughts and actions, as well as change, self-direction, stimulation, hedonism; (2) *conservation:* self-restriction, maintenance of traditional practices, protection of stability, security, conformity, tradition; (3) *self-enhancement:* sense and purpose of success and dominance over others: power and achievement, hedonism; and (4) *self-transcendence:* acceptance of others as equals and caring for the well-being of others: universalism and benevolence.

Fegg et al. (2014)^i^ found that, along the dimension "conservation" versus "openness to change", health care professionals working in maternity wards (MW-HCPs) ascribe higher importance to the values of "conformity" and "security", whereas health care professionals working in palliative care (PC-HCPs), ascribed higher importance to the values of "self-direction" and "stimulation". This difference was also observed on a dimensional level, with PC-HCPs preferring values in the value-dimension "openness to change" and MW-HCPs preferring values in the value dimension of "conservation". No differences amongst the samples were found in the “self” dimension. Furthermore, Fegg et al. (2014) indicate that, in comparing personal values, several differences emerged: PC-HCPs scored significantly higher in “openness to change” (comprising *self-direction*: e.g., freedom, creativity, being independent, being curious; *stimulation*: e.g., living an exciting and/or diversified life, being adventurous), whereas MW-HCPs scored significantly higher in “conservation” values (comprising *conformity*: e.g., courtesy, self-discipline, being respectful, being dutiful; *security*: e.g., social order, national security, feeling secure in family, being proper).

In Fegg et al. (2014), firstly, health care professionals report creativity and self-direction in their private and professional activities; secondly, they are excited, curious, and adventurous toward life – indicating a high level of experience values; thirdly, their focus on spirituality points out attitudinal values toward their life and professional work. Since the pioneering work done in the 1980s, the Schwartz value model has been studied, developed and researched worldwide and has become one of the most important VO models, particularly in educational and management contexts (Fromm, 2016; Louw, Mayer, & Baxter, 2012; Mayer, 2001; Schwartz & Bardi, 2001). However, it has hardly been used in therapeutic studies or to study FTTs development.

## Developing Value-Orientations in Family Therapist Trainees

Family therapy (FT) is based on the belief that the family is a unique social system with its own structure and patterns of communication and VOs have been well-researched in family members (Albert, Ferring, & Michels, 2013; Iruonagbe, Chiazor, & Foluke, 2013; Mert & Iskender, 2015). Each family develops its own unique personality, which is powerful and affects all of its members. According to Kluckhohn and Strodtbeck (1961), individuals’ value orientations influence their behaviour, conception and use of time; their relationships with their environment and with nature; and their interpersonal relationships. By focusing on the family’s value orientation, FTTs gain insight into the family’s view of the nature of the world and its problems, family member’s feelings about their problems, and the direction the family adopts to resolve problems (Ho, Rasheed, & Rasheed, 2004). However, the experience of differences in VOs in helping professions, as in FT, can lead to conflicts (Barsky, 2014), since values (Hecker, Trepper, Wetchler, & Fontaine, 1995) and value dilemmas can influence the therapy (Sanders, 2013).

Past research has shown that values in families and FT play a central role in terms of how the therapy develops and how the family might change through family therapy (Trotzer, 1981). Further, research has emphasised that client and therapist values influence the outcome of the therapy and that the client's responsiveness to the therapists' values influences symptoms, such as depression (Hamblin, Beutler, Scogin, & Corbishley, 1993). However, an increase in interest related to VO in therapies has occurred in recent years, particularly with regard to the central role of VOs in professional identity development (Brody & Doukas, 2014; Drolet & Désormeaux-Moreau, 2016; Iacobucci, Dal, Lindell, & Griffin, 2013) and the role that values play for the family therapist (Melito, 2003). Additionally, VOs influence the reasoning in therapy and in professional practice, in general (Fulford, Dickenson, & Murray, 2002; Seedhouse, 2005). VOs have been researched in psychotherapy and other forms of therapeutic and counselling interventions and contexts (Thomas, 1994; Vachon & Agresti, 1992). If health care professionals cannot work according to their values, they might develop burnout and problems in therapy (Beagan & Ells, 2009). Furthermore, strong value clashes and conflicts between therapist and client might even lead to strong ethical dilemmas and moral distress (Kälvemark, Haeıglund, Hansson, Westerholm, & Arnetz, 2004).

Fife and Whiting (2007) have highlighted that values play an important part in FT research and practice. However, many therapists seem to be relatively unaware about their own personal values and how their values affect their work. In their article, the authors highlight that marital and family therapy training should pay increased attention to values and their impact. This study takes the call for attention to values and their impact made by Fife and Whiting (2007) into account. To date, hardly any studies could be found that examine the values of FTTs within a longitudinal and developmental research setting in Germany. However, in this study it is assumed that values play an essential role for FTTs, to develop into their professional role and to build value-based client-therapist relationships. As highlighted by Drolet and Désormeaux-Moreau (2016, p. 273), for occupational therapists, the research on VOs can also help FTTs to "(i) facilitate client-centred approaches; (ii) foster inter-professional collaboration; (iii) reduce the ethical distress experienced by many healthcare professionals, (iv) improve both ethical professional practice and cultural awareness".

The purpose of this study is to respond to the need to gain an in-depth understanding of the VOs and their development in FTTs. The study was undertaken during a three-year training course in systemic family therapy (SFT) and using the well-researched value model of Schwartz as a theoretical and conceptual framework (Jambrak, Deane, & Williams, 2014; Schwartz & Bardi, 2001). The main aim of this study is to describe the development of VOs in a sample of FTTs during SFT. It thereby contributes to longitudinal psychological VO research in FTTs by responding to the research question: "What are the values of FTTs and how do they develop over the three-year professional training period?"

## Method

### Participants and Procedure

This study adopts a qualitative research design by using a longitudinal case study approach within the contemporary hermeneutical tradition (Bleicher, 2017; Dilthey, 2002). Since the research uses a context-specific standpoint (Patton, 2002), it requires an in-depth understanding of the current approach. The context of this research is a selected FTT centre in Germany and which is also a nationally accredited centre for FT training.

The study uses a longitudinal design to capture the development of VOs in FTTs throughout the three-year SFT course.

The sample was composed of FTTs registered for a three-year SFT course, at a private family therapy institution in Germany. The FTTs hold university degrees in Psychology, Social Work, Education or Social Sciences and are employed in related professions as, for example, psychologists, social workers, educators and consultants. Participants attend five to six course modules per year (the duration of each module is three to five days). Participants also meet in small peer-groups, between the facilitated modules, for supervision purposes and these meetings include exercises, reflection, discussions and interpersonal group coaching. Qualification criteria for participation are an A-level/Matric, a university degree and five years of professional/work experience in a relevant field of psychology, social work or education. Participation was not obligatory. All participants were enrolled for the three-year training course and, therefore, the entire population was targeted. All the FTTs completed the questionnaires at the beginning of each training year (three points of measurements). A few of the trainees discontinued the course of the training after year 1 or year 2.

The biographical data of FTTs show that, in the first year (2013), 66 individuals participated, while in 2014 and 2015, 55 and 43 individuals participated respectively. Participants stopped the training due to personal or professional reasons, such as change of place of residence, financial problems or illness. During the first year, out of the 65 individuals, 58 were German, one Polish, three Russian/German, one German/Persian, one German Swiss and one Croatian. Altogether, 12 participants were male, whilst 53 were female. The age range of the participants lay between 27 and 59 years. In year one, 33 were married, 23 single, 2 were living in partnerships, 5 were divorced, 2 were separated and 1 was widowed. One person spoke Croatian as a mother tongue, 5 were bilingual (3 German and Russian, 1 German and Persian, 1 German - Swiss German) and 59 participants spoke German as a mother tongue. Altogether, 35 participants were Protestant, 20 participants were Catholic, 1 was Lutheran, 1 was Greek-Orthodox, and 8 did not comment on religious affiliation. All the participants work in occupations within the social contexts (child care, social work, social education, counselling, family support/therapy/counselling/welfare or teaching).

### Instruments

At the commencement of each training year, participants were invited to complete two questionnaires.

1. The first questionnaire included closed-ended and open-ended questions of (1) a biographical and (2) a demographical nature in order to establish biographical and demographical insight. (The questions sought details of, for example, age, sex, professional background, mother tongue and occupation.)
2. The second questionnaire explored VOs. This questionnaire was developed on the basis of sound literature research and SFT, FTTs and VO. The questionnaire included questions, such as: "Please describe what VOs are important to you."; "What VO contribute to your health as a professional FTT?"; and "What are your personal interests and aims within the professional context?"

The participants completed the questionnaires on three separate occasions. The researchers used the same questionnaires throughout study in order to gain insight into the development of VO and, for example, changes in profession, family status and so on.

### Data Gathering and Data Analysis

Questionnaires were administered verbally, either by one of the researchers (during the first two years) or by the course facilitators (third year). A paper and pencil approach was applied. Data was analysed using the five-step process of content analysis (Terre Blanche, Durrheim, & Kelly, 2006, pp. 322–326): *familiarisation and immersion* – the researchers familiarised themselves with the gathered data, whilst integrating course observations; *inducing themes* – general themes in the data were defined; *coding* – categories and codes were inductively developed by identifying similarities and differences in the data; *elaboration* – categories and codes were further elaborated to enable understanding and reconstruction of the data; and *interpretation and checking to ensure data quality* – data were reconstructed and interpreted and set in a more complex context.

Frequencies with regard to codes and categories were analysed and statement frequency was interpreted according to the level of importance; for instance, the more often mentioned, the more important; the less often mentioned, the less important (as in Mayer, 2011). Frequencies were captured in tables and are presented in the findings section. Codes and categories emerged from the collected data and were compared (Chan, Walker-Gleaves, & Walker-Gleaves, 2015) with the SVM. Findings were interpreted in-depth, utilising the SVM. The data analysis used inductive and deductive processes and is therefore described as abductive (Mayer, 2011). Findings were validated through constant reflection and discussion amongst the researchers throughout the study period (Yin, 2009, p. 45).

Quality criteria, based on Lincoln and Guba's (1985) concept of trustworthiness, were taken into account. Further, the researchers aimed at establishing the quality of this research through performing systematic searches for and appraisals of research evidence (Richter Sundberg, Garvare, & Nyström, 2017). Similar criteria, such as confirmability, credibility, transferability and dependability were used to establish rigour and support trustworthiness (Shenton, 2004).

### Ethical Considerations

Ethical considerations include respect afforded to, and rights of, the interviewee, creation of informed consent, confidentiality, anonymity and transparency (Powell, Fitzgerald, Taylor, & Graham, 2012). Informed consent was provided by the participants and the SFT institution. Ethical clearance was provided by the European University Viadrina in Frankfurt (Oder), Germany, and by the SFT institution.

## Results

The following presentation of findings shows the VOs that were indicated in the questionnaires by the FTTs over a period of three years and in total. These VOs were then analysed and interpreted within the frame of the SVM. The tables in each section provide an overview of Vos, coded and clustered according to the SVM.

### Value-Orientations in Family Therapist Trainees Over Three Years

The most frequently mentioned value domain is "Benevolence", with 192 statements in the data set. "Benevolence" refers to the preservation and enhancement of the welfare of people with whom one is in frequent personal contact. FTTs work mainly with people and one major aim of FT is usually to preserve and/or enhance other people's welfare. Therefore, the high frequency of statements regarding "Benevolence" is not surprising. The values mentioned most frequently in this value domain are "honesty" (63 statements), "reliability" (35 statements), "friendship" (28 statements), "trust" (24 statements) and "sincerity" (11 statements). FTTs highlight honesty through, for example, this statement: "It is important for me to be honest when I work with my clients" (honesty). In terms of reliability, FTTs emphasise, for example: "I am a very reliable person and I like to work with people and help them." Another participant said: "In order to support people's well-being, you have to build trust in the relationship" (trust) and, "Sincerity is one of my most important values and it is also connected to honesty" (sincerity/honesty). The FTTs refer to the domain of "Benevolence", particularly with regard to the clients and in the development of the relationship between themselves as therapists and their clients.

**Table 2:** Schwartz Value Domain - Benevolence

Examples	Year 1	Year 2	Year 3	Total
Honesty	30	24	9	63
Reliability	12	14	9	35
Friendship	10	10	8	28
Trust	11	6	7	24
Sincerity	3	6	2	11
Helpfulness	4	3	-	7
Warm-heartedness	3	2	1	6
Empathy	4	1	-	5
Solidarity	3	2	-	5
Responsibility	3	-	1	4
Partnership	1	2	-	3
Inclusion	-	1	-	1

*Note.* Total for domain = 192.

Secondly, the value domain, "Self-direction", is referred to frequently in the data (see Table 3). The FTTs referred to a number of different values within that domain. Most mentioned was the value "freedom", with 16 statements, followed by "openness" (15 statements), "mindfulness" (11 statements) and "authenticity" (10 statements). All values mentioned decrease in number from year 1 to year 3 in their training, except the value of "freedom". It seems that FTTs value freedom particularly highly in year 3. One participant emphasises:

"*Freedom is so important to me. I want to live a free, self-directed life and be free in my decisions. That is very important to me*."

The increase in statements regarding the value of freedom might be due to the fact that FTTs have, by this point, come to understand the interrelationships within their families, their dependencies and their individual ways of individualisation and freedom from restrictions and dependencies. This development relates to the values, such as "self-respect", "prosperity", "compassion" and "optimism", which are frequently mentioned in year 3 (not before). One FTT explains, for example:

"*It is important to respect myself and to understand where I come from and why I am how I am. And I see now that sometimes, I thought I was free, but now I can see that I behaved like this, because of where I come from. I know I have to develop and to free myself and stay positive. The work with myself helps me in my profession*."

The high frequency of values in the domain "self-direction" shows the importance to FTTs of independent thought and action-choosing, creating and exploring, matters that are also important for the therapy practice. In some instances, the importance of being aware of the direction of the self is connected to a professional therapeutic practice and for FTTs to be clear about who they are and what they believe in order to become professional therapists. One participant mentions:

"*For growth I need to know what is important to me and I need to free myself from preoccupied thoughts and stereotypes*."

**Table 3:** Schwartz Value Domain - Self-direction

Examples	Year 1	Year 2	Year 3	Total
Freedom	1	7	8	16
Openness	3	8	4	15
Mindfulness	5	4	2	11
Authenticity	5	4	1	10
Optimism	2	1	3	6
Patience	1	2	2	5
Courage	2	2	-	4
Self-determination	2	1	-	3
Independence	2	-	1	3
Affectionate	2	-	-	2
Balance	-	2	-	2
Neutrality	1	-	-	1
Integrity	1	-	-	1
Self-consciousness	1	-	-	1
Curiosity	-	1	-	1
Education	-	1	-	1
Prosperity	-	-	1	1
Self-respect	-	-	1	1
Compassion	-	-	1	1

*Note.* Total for domain = 85.

The third most-mentioned domain is "Universalism", with 74 statements (see Table 4). "Universalism" relates to the understanding and appreciation of, as well as tolerance and protection for, the welfare of all people and for nature. As FTTs, participants need to relate to these universal values, particularly since they aim at understanding, appreciating and tolerating others. The values most frequently mentioned were "love" (33 statements), "tolerance" and "justice" (14 statements each), "acceptance" (4 statements), "equality" and "transparency" (3 statements each), "cohesion" (2 statements) and "humanity" (1 statement). Several participants emphasised statements such as, "Love is very important to me." Among the statements made by participants were:

"*Love is the basis to work with people. And only if I love, I can be tolerant."*

"When I have this deep love, then I can relate more easily to people."

"Tolerance! However, this is not always easy!"

"Love", as an overall approach to life and work, and within the work of FTTs, is the most important value, followed by "tolerance" and "justice", which are often integrated requirements in the profession of FT, where therapists work with individuals and families. FTs need to balance their own ideas and values when working with family systems with whom they might not share similar values and/or lifestyles.

**Table 4:** Schwartz Value Domain - Universalism

Examples	Year 1	Year 2	Year 3	Total
Love	15	11	7	33
Tolerance	6	7	1	14
Justice	5	3	6	14
Acceptance	-	2	2	4
Equality	2	1	-	3
Transparency	2	-	1	3
Cohesion	1	1	-	2
Humanity	1	-	-	1

*Note.* Total for domain = 74.

The value domain, "Tradition", was important for FTTs, particularly with regard to the values of "respect" (23 statements), "acknowledgement" (15 statements) and "faith" (14 statements) (see Table 5). "Respect" and "acknowledgement" are basic values with regard to ethical considerations when working in the FT contexts and were shown to be core values for FTTs. One participant explained: "I want to respect myself and others. That is not always easy, but I am learning."

It is interesting that faith is the only value which increases in frequency within the three-year training period. This might show that FTTs experience an increase in faith in terms of spirituality and, particularly, meaningfulness. All the other values mentioned decrease in frequency of being stated. Values such as "gratefulness", "hope", "commitment", "humbleness" and "ethics" are mentioned, but not very often.

One participant stated:

"*I need to believe in myself to be a good therapist. It is about believing and having faith ... in myself and in what I do ... and in the process*."

It can, however, be recognised that "humbleness" and "ethics", in particular, are mentioned only from year 2 onwards, for instance,

"*I want to behave in an ethical manner" or "To become humble is important to me ... humbleness ... and to be grateful*".

This might relate to the increase of faith (including meaningfulness): FTTs experienced an increase in ethical values and humbleness towards their own processes and their life.

**Table 5:** Schwartz Value Domain - Tradition

Examples	Year 1	Year 2	Year 3	Total
Respect	14	6	3	23
Acknowledgement	8	4	3	15
Faith	3	5	6	14
Hope	1	4	1	6
Humbleness	-	2	1	3
Gratefulness	1	1	-	2
Ethics	-	-	2	2
Commitment	1	-	-	1

*Note.* Total for domain = 66.

The value domain of "Conformity" (41 statements) closely follows the number of statements in respect of the value domain of "Tradition" (see Table 6). Here, the most important value refers to the "family" (25 statements). The family is referred to as an important value (e.g. "Family is very important to me."), in terms of honouring family members and in terms of not violating any social expectations or norms relating to the family. Other values which fall into this value domain are "punctuality", "loyalty" and "harmony" (4 statements each). Finally, in 2 statements, "belonging" is mentioned, as are "discipline" and "politeness", with 1 statement each. The data show that the family is particularly important with regard to the value domain of "conformity". This is not surprising, particularly because FTTs are very aware of family issues and are sensitive regarding family relationships. This connection is, for example, expressed in statements, such as:

"*Family. I think you must value people in the family and I just like to be with my family and do something with my family*."

**Table 6:** Schwartz Value Domain - Conformity

Examples	Year 1	Year 2	Year 3	Total
Family	14	8	3	25
Punctuality	2	1	1	4
Loyalty	1	2	1	4
Harmony	1	2	1	4
Belonging	1	-	1	2
Discipline	1	-	-	1
Politeness	-	1	-	1

*Note.* Total for domain = 41.

The value domain of "Hedonism" is one that is not mentioned frequently in the data set (see Table 7). Examples of values referred to in the context of "Hedonism" are "happiness" (10 statements), "contentedness" (9 statements) statements, and "joy of life" (4 statements), "humour" (3 statements) and "fulfilled life" (1 statement). This shows that, within the domain "Hedonism", mainly "happiness" and "contentedness" are important for FTTs over the three-year period of training and are expressed as follows:

"*I want to be happy and content in my life and in my work. That is important. If I am not happy, I should change.*"

**Table 7:** Schwartz Value Domain - Hedonism

Examples	Year 1	Year 2	Year 3	Total
Happiness	5	2	3	10
Contentedness	4	5	-	9
Joy of life	-	2	2	4
Humour	1	1	1	3
Fulfilled life	-	-	1	1

*Note.* Total for domain = 27.

The domain "Security" is one of the domains not frequently mentioned by FTTs (see Table 8). "Security", if mentioned at all, is related mainly to health and the feeling of security due to being healthy and feeling healthy (9 statements):

"*I think the environment needs to be safe and secure. Then you can feel healthy and connected and peaceful and this is a good basis*."

Seven statements refer to security in terms of "peace", "peacefulness" and "calmness". Six statements refer to "safety" and only three statements highlight the importance of "continuity", mainly referring to the experiences of continuous learning and continuing positive experiences for example in a family or during the socialisation and enculturation processes.

"*I feel secure when I have got long-term relationships, stable and enduring relationships which are good for me*."

With 25 statements altogether, "security" is one of the least mentioned value domains in this sample, probably due to the fact that it can be assumed that the participants belonged to the middle or higher class in society. All participants held a university degree and most of the participants were German-speaking German citizens and, like a majority of Germans, had been born and raised in middle or upper-class contexts within Germany. It might be assumed that security is important to them, but it is not mentioned more frequently, due to the fact that it is seen as "a given" and, therefore, hardly mentioned in this research as an important value domain. It is also interesting that only female FTTs refer to the value domain of "security".

**Table 8:** Schwartz Value Domain - Security

Examples	Year 1	Year 2	Year 3	Total
Health	2	4	3	9
Peace	4	3	-	7
Safety	2	4	-	6
Continuity	1	2	-	3

*Note.* Total for domain = 25.

The value domain, "Achievement", was mentioned only in the context of seven statements and it is highlighted that this was particularly in years 1 and 2 (see Table 9). This means that, only in year 1 and 2, is "work" emphasised as an important value in 4 statements, whilst "professionalism" is highlighted in one statement each in the first two years and "effectiveness" as an important work value is mentioned once in year 2. This shows that the value domain, "Achievement", is less important as a value domain for FTTs and that the meaning of the profession seems not to be related to the value of "achievement" but seems rather to be related to other value sets, as mentioned above. Exemplary values, such as "success", "capability" and "ambition", as mentioned by Schwartz (1994), are not reported at all in the data, which shows that this sample does not relate to these values supporting the domain of "Achievement".

**Table 9:** Schwartz Value Domain - Achievement

Examples	Year 1	Year 2	Year 3	Total
Work	3	1	-	4
Professionalism	1	1	-	2
Effectiveness	-	1	-	1

*Note.* Total for domain = 7.

The two value domains mentioned least frequently by FTTs are "stimulation" and "power" (see Table 10). One person, in year 2, emphasised "excitement" as an important value, stating:

"*I like it when I become excited and when I can start something new with excitement and learn*."

Only one person in year 2 mentioned "wealth" as an important value in the value domain of "power". Interestingly, the value domains, "stimulation" and "power" were highlighted by male participants. No female participant referred to the categories "stimulation" and "power".

**Table 10:** Schwartz Value Domains - Stimulation and Power

Examples	Year 1	Year 2	Year 3	Total
Excitement	-	-	1	1
Wealth	-	1	-	1

*Note.* Total for domain = 2.

The findings show that, with regard to the four value dimensions mentioned in Schwartz (1994, p. 24–25), FTTs mainly referred to value domains within the value-dimension of "self-transcendence" (266 statements), followed by the value domains within the value-dimension of "conservation" (109 statements), "openness to change" (86 statements) and, finally, the value domains within the value dimension of "self-enhancement" (35 statements) (see Table 11).

**Table 11:** Value Dimensions

Value dimension / Value domains	Frequencies
openness to change	**86**
self-direction (independent thoughts and actions)	85
stimulation (change)	1
conservation	**109**
security (protection of stability)	25
conformity (self-restrictions)	41
tradition (maintenance of traditional practices)	43
self-enhancement	**35**
power (dominance)	1
achievement (success)	7
hedonism (happiness)	27
self-transcendence	**266**
universalism (acceptance of others as equals)	74
benevolence (caring for the well-being of others)	192

This shows that caring for the well-being of others and acceptance of others as equals are highly important for FTTs, which is probably an advantage when working in therapeutic or social professions, as FTTs do. Further on, the second highest value domain of "self-direction" might also help FTTs to find their own, independent way of working with their clients in the value dimension of "openness to change". The second most frequently mentioned value dimension of "conservation" shows that values such as family, along with the applying of self-restrictions in order to fit in with the social norms and security/stability are highly important for FTTs, something that is probably reflected in the fact that they work primarily with families, the traditional core unit of the nation state. For FTTs, self-enhancement and their careers, their power and achievement are at least frequently mentioned and therefore interpreted as being of less importance. This fact, however, also includes the potential danger that FTTs might forget about themselves and their own, personal well-being whilst working with clients, by focusing on the well-being of the clients only.

## Discussion

This study stands in the tradition of value research within the psychological discipline (Jambrak, Deane, & Williams, 2014; Rokeach, 1973; Schwartz, 1994), focusing on educational and training contexts (Boness, 2002; Patel, 2016) and responding to the aim of describing the development of VOs in FTTs. The study also supports the idea that VOs are dynamic, change over time and vary in importance (Gouveia, Vione, Milfont, & Fischer, 2015; Schwartz, 1994). According to Kluckhohn and Strodtbeck (1961), values comprise needs of individuals as biological organisms; requisites of coordinated, social and interpersonal interaction; and security of functions concerning the well-being and the survival of groups. This study shows that the focus of FTTs, in terms of values, is based in the form of requisites of coordinated, social and interpersonal interactions.

Findings in this study show the frequency of VOs (Schwartz, 1994; Schwartz & Bilsky, 1987) – from most mentioned to least mentioned – to be: "Benevolence", followed by "Self-direction", "Universalism", "Tradition", "Conformity", "Hedonism", "Security", "Achievement", …"Stimulation" and "Power" (that is, power to regulate social interactions and behaviours) and all viewed from the perspective of FTTs. The value-dimensions (Schwartz, 1994; Schwartz & Bilsky, 1987) most frequently mentioned in this study, such as: "self-transcendence", "conservation, "openness to change" and "self-enhancement" show that, in terms of differentiation (Oishi et al., 1998), collectivist values ("self-transcendence", "conservation") seem to be more frequently mentioned and therefore more important for FTTs than individualistic values ("openness to change" and "self-enhancement"). This assumes that the value dimensions concerned are viewed as overall categories of value domains and values.)

The focus on the social realm, the system, as the family, seems to be more important than the focus on the individual. The systemic perspective is therefore inherent in the value domains and dimensions mentioned.

The study does not provide information on the dialectics and contradictions of VO, as highlighted in previous research such as that of Hiratsuka, Suzuki, and Pusina (2016), or conflictual outcomes of differences in value-sets as described previously (Barsky, 2014; Berkel, 2005; Janssen & Van Berkel, 2015). Rather, it provides a descriptive, harmonic and integrated view on values over the three-year period. FTTs seem to be quite conscious and aware of their values and do not highlight their contradictions and dialectics, but rather present a harmonic and integrated value concept.

The VO described by FTTs in the study seem to fit positively with the development of the professional identity (Brody & Doukas, 2014; Drolet & Désormeaux-Moreau, 2016; Iacobucci, Dal, Lindell, & Griffin, 2013; Levy, Shlomo, & Itzhaky, 2014) of the FTT, taking the therapist-client relationship and treatment outcomes into account (Huang & Zane, 2016) and reflecting the strong motivation of FTTs to develop within their new field of training and expertise, contributing to general mental health and well-being (Jambrak, Deane, & Williams, 2014).

This study does not support the findings by Fegg et al. (2014) in terms of preferences in VO of HCPs, since FTTs score highest in "self-transcendence", whilst HCPs prefer "conservation" and "openness to change". Moreover, this study does not provide any information on how the FTT values and value contradictions or conflicts influence the client and the client's system or symptoms, as described previously (Barsky, 2014; Hecker, Trepper, Wetchler, & Fontaine, 1995; Sanders, 2013).

Since, to date, hardly any studies could be found which examine the values of FTTs within a longitudinal and developmental research setting, either internationally or in Germany, only a few similar previous studies were available with which to compare the data. In comparison to occupational therapists (Drolet & Désormeaux-Moreau, 2016, p. 273), the findings this study presents for FTTs show that Vos have to be understood, particularly in terms of the FTTs’ interest and in order to facilitate client-centred approaches ("Benevolence", "Universalism", "Tradition", "Conformity") as well as to foster inter-professional collaboration ("Achievement"). Reducing the ethical distress experienced by many HCPs and improving both an ethical professional practice and a cultural awareness (as mentioned by Drolet and Désormeaux-Moreau, 2016 for HCP) are not mentioned by FTTs in terms of their VOs. Finally, it can be concluded that the FTTs studied in this research are well aware of their values as opposed to being unaware to the large extent emphasised by Fife and Whiting (2007) in their research on FTTs.

### Limitations of This Study

The brevity of the discussion of findings within the context of existing literature shows that the findings should be viewed and interpreted against certain limitations of the study. The first limitation relates to the limited and explorative nature of the research as the researchers had access to only three sets of data over a period of three years. Secondly, the study is limited to a relatively small sample size. Thirdly, the study is limited with regard to the demographic and biographic composition of the sample, a clear cultural and religious bias (most of the participants are female, German and Christian). The study should therefore be replicated in different contexts and cultures and compared with the VO of FTTs in different countries. Fourthly, only a limited number of questions were asked in the questionnaire and no interviews could be conducted to explore the responses in more depth. Therefore, the depth of the statements is limited and could not be explored more deeply. Finally, the study's findings could be discussed only in the context of a limited literature review in terms of the specific context and setting of the study. This study is, therefore, clearly of an explorative nature.

### Conclusions and Recommendations

This study aimed at exploring and understanding the development of VOs in FTTs over a three-year training course period. They show that FTTs focus mainly on VOs in the value domain of "benevolence", followed by "self-direction" and "universalism" and are therefore anchored in collectivist values rather than in individual ones. The least mentioned value domains include "power" and "stimulation". With regard to the Schwartz value dimensions, the dimension of "self-transcendence" is most frequently mentioned, followed by "conservation", "openness to change" and "self-enhancement". This shows that FTTs’ VOs relate strongly to caring for the well-being of others, independent thought, action and acceptance of others as equals, and are therefore particularly socially directed.

Over the three-year period, minor value shifts were observed, specifically an increase in value statements such as "freedom" (self-direction) – which might relate to an increased understanding of personal family dependencies across the training period, and an increase in the value frequencies of "justice" (universalism), as well as "faith" (in terms of spirituality/meaningfulness), in the value domain of "tradition". All other VOs showed a decrease in the frequency of statements over the three-year period. With regard to future research, further investigations are needed and these should explore VOs and their change in FTTs in different institutions across a three-year training period. Studies should use mixed methods to explore the VOs, based on quantitative and qualitative methodological approaches, with this being achieved by using questionnaires as well as in-depth interviews. Follow-up studies with the FTTs should be conducted after they have worked as FTs, while VOs should be compared between FTTs and FTs, who have been in practice for a certain amount of time.

On a practical note, the development of VOs in FTTs should become a conscious process during the three-year training period. Trainers and FTTs need to increase their awareness regarding their VOs and the changes they experience with regard to their personal and professional development in terms of VOs. VOs could be consciously developed through self-reflections in the context of personality development and professionalism and the connection between the VO, the shift in the VO and the behavioural patterns of FTTs should be explored to understand how VOs (shifts) influence and are influenced by behavioural patterns. VOs could be explored during the training through self-reflection, discussions in small groups, exercises, and the learning unit regarding the development of ethical behaviour in FT. Trainers in FT institutes also need to develop their awareness regarding the development of VOs and make it a conscious part within FT training, which influences and informs behaviour patterns which are established in FTTs during the three-year training course.

## References

[r1] Alazaizeh M. M. Hallo J. C. Backman S. J. Norman W. C. Vogel M. A. (2016). Value orientations and heritage tourism management at Petra Archaeological Park Jordan. Tourism Management, 57, 149–158. 10.1016/j.tourman.2016.05.008

[r2] Albert I. Ferring D. Michels T. (2013). Intergenerational family relations in Luxembourg: Family values and intergenerational solidarity in Portuguese immigrant and Luxembourgish families. European Psychologist, 18(1), 59–69. 10.1027/1016-9040/a000125

[r3] Barsky, A. E. (2014). *Conflict resolution for the helping professions* (2nd ed.). Oxford, United Kingdom: Oxford University Press.

[r4] Beagan B. Ells C. (2009). Values that matter, barriers that interfere: The struggle of Canadian nurses to enact their values. Canadian Journal of Nursing Research, 41, 86–107. 19485047

[r5] Berkel K. (2005). Wertkonflikte als Drama – Reflexion statt Training. Wirtschaftspsychologie, 4, 62–70.

[r6] Bleicher, J. (2017). *Contemporary hermeneutics.* New York, NY, USA: Routledge.

[r7] Bond, M. H. (1998). *Social psychology across cultures* (2nd ed.). Hertfordshire, United Kingdom: Prentice Hall.

[r8] Boness, C. (2002). *Kritische Situationen in Begegnungen zwischen Tansaniern und Europäern: Eine Felduntersuchung im Sekundarschulsystem Tansanias* (Europäische Hochschulschriften, Reihe 11: Pädagogik, Vol. 859). Frankfurt (Main), Germany: Peter Lang.

[r9] Brody H. Doukas D. (2014). Professionalism: A framework to guide medical education. Medical Education, 48, 980–987. 10.1111/medu.12520 25200018

[r10] Chan N. N. Walker-Gleaves C. Walker-Gleaves A. (2015). An exploration of students’ lived experiences of using smartphones in diverse learning contexts using a hermeneutic phenomenological approach. Computers & Education, 82, 96–106. 10.1016/j.compedu.2014.11.001

[r11] Dilthey, W. (2002). The formation of the historical world in the human sciences. In R. A. Makkreel & F. Rodi (Eds.), *Wilhelm Dilthey: Selected Works: Volume 3. The formation of the historical world in the human sciences* (pp. 416–431). Princeton, NJ, USA: Princeton University Press. (Original work published 1910)

[r12] Drolet M.-J. Désormeaux-Moreau M. (2016). The values of occupational therapy: Perceptions of occupational therapists in Quebec. Scandinavian Journal of Occupational Therapy, 23(4), 272–285. . 10.3109/11038128.2015.1082623 27215136

[r13] Druckmann D. Broom B. J. Korper S. H. (1988). Value differences and conflict management: Facilitation or delinking? The Journal of Conflict Management, 32, 234–251.

[r14] Edwards A. W. (2014). Therapeutic values clarification and values development for end-of life patients: A conceptual model. The American Journal of Hospice & Palliative Care, 31(4), 414–419. 10.1177/1049909113486337 23661769

[r15] Fegg M. L’hoste S. Brandstatter M. Borasio G. D. (2014). Does the working environment influence health care professionals’ values, meaning in life and religiousness? Palliative care units compared with maternity wards. Journal of Pain and Symptom Management, 48(5), 915–923. . 10.1016/j.jpainsymman.2014.01.009 24727306

[r16] Fife S. T. Whiting J. B. (2007). Values in family therapy practice and research: An invitation for reflection. Contemporary Family Therapy, 29(1-2), 71–86. 10.1007/s10591-007-9027-1

[r17] Fromm, E. (2016). *Psychologie und Werte: Values, psychology, and human existence* Munich, Germany: Open Publishing Rights GmbH. (Original work published 1959 in A. Maslow [Ed.], *New knowledge in human values*, New York, NY, USA: Harper & Bros.).

[r18] Fulford, K. W. M., Dickenson, D. L., & Murray, T. H. (2002). Many voices: Human values in healthcare ethics. In K. W. M. Fulford, D. L. Dickenson, & T. H. Murray (Eds.), *Healthcare ethics and human values: An introductory text with readings and case studies* (pp. 1-19). Oxford, United Kingdom: Blackwell.

[r19] Gouveia V. V. Vione K. C. Milfont T. L. Fischer R. (2015). Patterns of value change during the life span: Some evidence from a functional approach to values. Personality and Social Psychology Bulletin, 41(9), 1276–1290. 10.1177/0146167215594189 26187119

[r20] Hamblin D. Beutler L. Scogin F. Corbishley A. (1993). Patient responsiveness to therapist values and outcome in group cognitive therapy. Psychotherapy Research, 3(1), 36–46. 10.1080/10503309312331333649

[r21] Hecker L. L. Trepper T. S. Wetchler J. L. Fontaine K. L. (1995). The influence of therapist values, religiosity and gender in the initial assessment of sexual addiction by family therapists. The American Journal of Family Therapy, 23(3), 261–272. 10.1080/01926189508251356

[r22] Hiratsuka H. Suzuki H. Pusina A. (2016). Explaining the effectiveness of the contrast culture method for managing interpersonal interactions across cultures. Journal of International Students, 6(1), 73–92.

[r23] Ho, M. K., Rasheed, J. M., & Rasheed, M. N. (2004). *Family therapy with ethnic minorities* (2nd ed.). Thousand Oaks, CA, USA: Sage.

[r24] Howell, A. J., & Larsen, D. J. (2015). Other-oriented hope reflects on orientation toward others. In *Understanding other-oriented hope: An integral concept within hope studies* [SpringerBriefs in Well-Being and Quality of Life Research] (pp. 7–17). 10.1007/978-3-319-15007-9_2

[r25] Huang C. Y. Zane N. (2016). Cultural influences in mental health treatment. Current Opinion in Psychology, 8, 131–136. 10.1016/j.copsyc.2015.10.009 29506788 PMC9528809

[r26] Iacobucci T. A. Dal B. J. Lindell D. Griffin M. Q. (2013). Professional values, self-esteem, and ethical confidence of baccalaureate nursing students. Nursing Ethics, 20, 479–490. 10.1177/0969733012458608 23166146

[r27] Iruonagbe T. C. Chiazor I. A. Foluke A. (2013). Revisiting family values: A pathway towards societal stability. Gender & Behaviour, 11(2), 5635–5642.

[r28] Jambrak J. Deane F. P. Williams V. (2014). Value motivations predict burnout and intentions to leave among mental health professionals. Journal of Mental Health, 23(3), 120–124. 10.3109/09638237.2013.869576 24433193

[r29] Janssen F. J. J. M. Van Berkel B. (2015). Making philosophy of science education practical for science teachers. Science & Education, 24(3), 229–258. . 10.1007/s11191-014-9735-5

[r30] Kälvemark S. Haeıglund A. T. Hansson M. G. Westerholm P. Arnetz B. (2004). Living with conflicts-ethical dilemmas and moral distress in the health care system. Social Science & Medicine, 58(6), 1075–1084. 10.1016/S0277-9536(03)00279-X 14723903

[r31] Kluckhohn, F. R., & Strodtbeck, F. L. (1961). *Variations in value orientations*. Evanston, IL, USA: Row, Peterson.

[r32] Levy D. Shlomo S. B. Itzhaky H. (2014). The ‘building blocks’ of professional identity among social work graduates: Social work education. International Journal, 33(6), 744–759.

[r33] Lincoln, Y. S., & Guba, E. G. (*1985*). *Naturalistic inquiry*. Newbury Park, CA, USA: Sage.

[r34] Louw L. Mayer C.-H. Baxter J. (2012). Exploring relationship between value- and life-orientation and job satisfaction. Acta Commercii, 12, 45-67.

[r35] Mayer, C.-H. (2001). *Werteorientierungen an Sekundarschulen in Tanzania vor dem Hintergrund interkultureller afrikanischer Wertediskussionen*. Stuttgart, Germany: Ibidem.

[r36] Mayer, C.-H. (2008). *Managing conflict across cultures, values and identities: A case study in the South African automotive industry*. Marburg, Germany: Tectum Verlag.

[r37] Mayer, C.-H. (2011). *The meaning of sense of coherence in transcultural management.* Münster, Germany: Waxmann.

[r38] Mayton D. M. II Ball-Rokeach S. J. Loges W. E. (1994). Human values and social issues: An introduction. The Journal of Social Issues, 50(4), 1–8. 10.1111/j.1540-4560.1994.tb01194.x

[r39] Melito R. (2003). Values in the role of the family therapist: Self-determination and justice. Journal of Marital and Family Therapy, 29(1), 3–11. 10.1111/j.1752-0606.2003.tb00378.x 12616794

[r40] Mert A. Iskender M. (2015). Systemic family-oriented program of psycho-education, effect of values of spouses and perceived social support on dyadic adjustment. International Journal of Human and Behavioral Science, 1(3), 1–12. 10.19148/ijhbs.81297

[r41] Oishi S. Schimmack U. Diener E. Suh E. M. (1998). The measurement of values and individualism-collectivism. Personality and Social Psychology Bulletin, 24(11), 1177–1189. 10.1177/01461672982411005

[r42] Patel, P. (2016). Cultural value orientations and employee preference for HRM practices in Northern European countries: A research agenda on the Swedish perspective. In *Proceedings of the 2016 Irish Academy of Management Annual Conference, Dublin, Ireland**.*

[r43] Patton, M. Q. (2002). *Qualitative research and evaluation methods*. Thousand Oaks, CA, USA: Sage.

[r44] Powell, M. A., Fitzgerald, R. M., Taylor, N., & Graham, A. (2012). *International literature review: Ethical issues in undertaking research with children and young people* [Dunedin, New Zealand: Southern Cross University ePublications@SCU]. Retrieved from https://epubs.scu.edu.au/ccyp_pubs/40/

[r45] Richter Sundberg L. Garvare R. Nyström M. E. (2017). Reaching beyond the review of research evidence: A qualitative study of decision making during the development of clinical practice guidelines for disease prevention in healthcare. BMC Health Services Research, 17, 344. 10.1186/s12913-017-2277-1 28490325 PMC5426017

[r46] Rokeach, M. (1973). *The nature of human values.* New York, NY, USA: Free Press.

[r47] Rusk R. D. Waters L. (2015). A psycho-social system approach to well-being: Empirically deriving the five domains of positive functioning. The Journal of Positive Psychology, 10(2), 141–152. . 10.1080/17439760.2014.920409

[r48] Sanders, R. K. (2013). *Christian counseling ethics: A handbook for psychologists, therapists and pastors* (2nd ed.). Downers Grove, IL, USA: Intervarsity Press.

[r49] Schwartz S. H. (1994). Are there universal aspects in the structure and content of human values? The Journal of Social Issues, 50(4), 19–45. 10.1111/j.1540-4560.1994.tb01196.x

[r50] Schwartz S. H. (2012). An overview of the Schwartz value theory of basic values. Online Readings in Psychology and Culture, 2(1), 11 10.9707/2307-0919.1116

[r51] Schwartz S. H. Bardi A. (2001). Value hierarchies across cultures taking a similarities perspective. Journal of Cross-Cultural Psychology, 32(3), 268–290. 10.1177/0022022101032003002

[r52] Schwartz S. H. Bilsky W. (1987). Toward a universal psychological structure of human values. Journal of Personality and Social Psychology, 53(3), 550–562. 10.1037/0022-3514.53.3.550

[r53] Seedhouse, D. (2005). *Values-based decision-making for the caring professions.* New York, NY, USA: Wiley.

[r54] Shenton A. K. (2004). Strategies for ensuring trustworthiness in qualitative research projects. Education for Information, 22, 63–75. 10.3233/EFI-2004-22201

[r55] Sinding, K., Waldstrom, C., Kreitner, R., & Kinicki, A. (2014). *Organisational behaviour* (5th ed.). Berkshire, United Kingdom: McGraw-Hill Education.

[r56] Terre Blanche, M., Durrheim, K., & Kelly, K. (2006). First steps in qualitative data analysis. In M. Terre Blanche, K. Durrheim, & D. Painter (Eds.), *Research in practice: Applied methods for the social sciences* (pp. 321–344). Cape Town, South Africa: University of Cape Town.

[r57] Thomas V. (1994). Value analysis: A model of personal and profession ethics in marriage and family counseling. Counseling and Values, 38, 193–204. 10.1002/j.2161-007X.1994.tb00837.x

[r58] Trotzer J. P. (1981). The centrality of values in families and family therapy. International Journal of Family Therapy, 3(1), 42–55. 10.1007/BF00936269

[r59] Vachon D. O. Agresti A. A. (1992). A training proposal to help mental health professionals clarify and manage implicit values in the counseling process. Professional Psychology, Research and Practice, 23, 509–514. 10.1037/0735-7028.23.6.509

[r60] Yin, R. K. (2009). *Case study research: Design and methods* (4th ed.). London, United Kingdom: Sage.

